# The Medico-Psychological Association and Their Nurses

**Published:** 1897-02-06

**Authors:** 


### Editor's Letter-Box.

[Our Correspondents are reminded that prolixity is a great bar to publication, and that brevity of style and conciseness of statement greatly facilitate early insertion.]

### THE MEDICO-PSYCHOLOGICAL ASSOCIATION AND THEIR NURSES.

"One of the Public" writes: Will you allow me of your courtesy to protest, as one of the public, against the assertion of Dr. Ernest White that "The public is at last realising that the hospital nurse of to-day is over-educated and a possible source of danger." To whom—the doctors or the public? If the latter, allow me to correct him. No, sir, the danger is not in nurses being too well educated and trained, but in undertrained and careless doctors. I can understand doctors who have, possibly to their own, and certainly to their fellow student's astonishment, scrambled through their examinations and obtained their diplomas, disapproving of having a skilled nurse of the present day overlooking and mentally criticising their unskilful treatment of a disease. But for the life of me I cannot see where the "danger to the public" comes in in thus being safeguarded.

DR. ERNEST WHITE, City of London Asylum, writes: Your correspondent, who shelters himself under the initials "M.D." in your issue of the 30th ult., is obviously ignorant of asylum life and the training of mental nurses. He states the mental nurse requires three years' training. Permit me to inform him that two years is the period fixed by the Medico-Psychological Association. He re-echoes your sentiments condemnatory of the absurd and preposterous idea that responsible nursing posts in asylums should only be given to those who hold the certificate of the Medico-Psychological Association. If he only knew the requirements necessary for such posts he would understand that the possession of this certificate is essential. It represents a certain standard of efficiency, as proved by training and examination, which I maintain ordinary nursing certificates do not. It differs as widely from many of the latter as our medical qualifications, capable of registration, do from the easily obtained degrees of the United States. Hospitals may train and certificate their own nurses, either without or after examination, some hospitals turning out good nurses, others bad, but uniformity of training and examination is the only safeguard to the public, and sooner or later this will be essential for the hospital as well as the asylum nurse. It should be noted that the Medico-Psychological Association distinctly encourages the hospital-trained nurse to become a mental nurse by admitting her to its examination after one year's residence in an asylum, and this is certainly, for those who desire to undertake this branch of work, a step in the right direction, but I must reiterate that in the future no post of responsibility in the wards of an asylum (such as charge attendant or charge nurse) will be given to anyone who does not possess the nursing certificate of the Medico-Psychological Association.

DR. OUTTERSON WOOD writes: I have been glad to observe how, up to now, mental nurses have avoided the trap so temptingly laid for them in the columns of one of your contemporaries, and I desire to offer a friendly word of warning to your correspondent, "An Old Nurse," who writes to you on the subject of the Medico-Psychological Association and its nurses. I would ask her to disregard such ridiculous statements as "forcing mental nurses to join the R.B.N.A." and "degradation to hospital nurses," &c., &c. These statements have been made for the purpose of arousing among mental nurses and others a feeling of indignation against and opposition to the R.B.N.A., but I would point out they have not been made by responsible persons who have the welfare of nurses and the R.B.N.A. at heart. I wish to assure your correspondent the Medico-Psychological Association has not made "such a blunder as to try and force asylum nurses on to the R.B.N.A.'s register." It has taken no such action. There is no "fighting and throwing mud." Asylum superintendents *do* train their own staff, and they require no extra medical officers to give lectures, for they are already given by the existing medical staff as part of their duty. There is no such policy adopted as "forcing mental nurses on a register other than their own." I claim that all qualified nurses, medical or mental, surgical or obstetric, or fever, or any other class, have "a right of entry" to the published register of the R.B.N.A., and that it has full power, and indeed it is in accordance with the letter and the spirit of its charter to admit them. I challenge any proof to the contrary. It is no insult to any department of nursing that all should be banded together in one Royal Nursing Association, whatever their specialty may be, each having a separate department in the register, and assuredly that time will come. I am confident it is not the hospital nurses themselves who resist the claims of mental nurses. It is the narrow-minded policy of a few who would restrict the aims and scope of the R.B.N.A., and who are striving to stir up hospital nurses to oppose mental nurses for that purpose. It is with the view of defeating such an unworthy object that I ask you to kindly give me space in your paper to warn mental nurses against being led away by self-interested and inaccurate statements. Nurses of all classes owe you a debt of gratitude for having placed your valuable space at their disposal. I am positive the more this subject is ventilated, the more will we find properly trained and qualified nurses of all classes joining hands in one common sisterhood to resist the encroachments of the unqualified, who are usurping their functions and defrauding the public. I maintain it is the function and the duty of the R.B.N.A. to aid all classes of qualified nurses by placing them on its register; by so doing it will help to secure them against fraudulent imitation, and afford protection to the public, which is the very *raison d'être* of its existence.

